# Monash Health Butterfly Day Program Parent/Carer Group

**DOI:** 10.1186/2050-2974-3-S1-P17

**Published:** 2015-11-23

**Authors:** Joanne Webb, Giuseppina Pompei

**Affiliations:** 1 Monash Health Butterfly Day Program, Lelbourne, Australia

## Background/purpose

The NICE guidelines (2004) emphasise the importance of involving family members in eating disorder treatment. However, following an internal review of the family and carer involvement of individuals at the Butterfly Day Program (BDP), it was recognised that the Program would benefit from offering increased family support when treating young people with an eating disorder.

## Aims

The BDP Parent/Carer group aims to better include parents/carers in the program by increasing understanding of the Program; providing information and support specific to the group therapy offered at BDP; and by providing an opportunity for parents/carers to share their experiences with other parents/carers.

## Method

Groups were held three times a year, and each group was structured in its delivery. Groups were facilitated by program clinicians.

## Results

The Outcome Rating Scale (Miller et al., 2003) was adapted for the nature of the groups offered to each parent/carer to complete at the end of each session. The self-report data indicated high parent/carer satisfaction with feeling validated in the group, group content, presentation, and relevance. Additional qualitative data further indicated strong parent satisfaction, particularly regarding group content, parent support and an opportunity to meet and share with other parents.

## Conclusions

A valued service initiative that has been well received by parents. However, further development needed to best meet the needs of the current population and to increase group attendance.

